# Overcoming Immune Evasion in the Prostate Tumor Microenvironment: Novel Targeted Strategies to Improve Treatment Outcomes

**DOI:** 10.3390/cancers17213441

**Published:** 2025-10-27

**Authors:** Jing Huang, Ademola Ojo, Serena Tsao, Amir Horowitz, Natasha Kyprianou, Che-Kai Tsao

**Affiliations:** 1 Tisch Cancer Institute, Icahn School of Medicine at Mount Sinai, New York, NY 10029, USA; ademola.ojo@mountsinai.org (A.O.); serenatsao09@gmail.com (S.T.); amir.horowitz@mssm.edu (A.H.); natasha.kyprianou@mountsinai.org (N.K.); 2 Department of Immunology & Immunotherapy, Lipschultz Precision Immunology Institute, Icahn School of Medicine at Mount Sinai, New York, NY 10029, USA; 3 Department of Oncological Sciences, Icahn School of Medicine at Mount Sinai, New York, NY 10029, USA; 4 Department of Urology, Icahn School of Medicine at Mount Sinai, New York, NY 10029, USA; 5 Northwell Cancer Institute, New Hyde Park, New York, NY 11042, USA; ctsao@northwell.edu

**Keywords:** prostate cancer, tumor microenvironment, metastatic castration-resistant prostate cancer, biomarker-driven treatment, immune evasion

## Abstract

Despite advances in diagnostic and therapeutic technology, prostate cancer remains a leading cause of morbidity and mortality among men. While androgen deprivation therapy and next-generation androgen receptor pathway inhibitors offer durable responses, the emergence of the lethal phenotype, metastatic castration-resistant prostate cancer (mCRPC) eventually develops for most. A growing body of evidence points to the tumor microenvironment (TME) as a key driver of immune evasion and therapeutic failure. This review focuses on the current knowledge of immune suppression in the prostate TME, including cancer-associated fibroblasts, myeloid-derived suppressor cells, tumor-associated macrophages, immune checkpoint pathways, and several associated key metabolic alterations. These cellular and molecular networks contribute to therapeutic resistance and disease progression and may be used as therapeutic targets. We will also examine emerging treatment strategies aimed at reprogramming the TME, as well as combination approaches incorporating immunotherapies with other signaling inhibitors. Future success in clinical therapeutic development for mCRPC will depend on rational combinations that address both tumor-intrinsic resistance and extrinsic immune suppression, with emphasis on biomarker-driven patient and treatment selection.

## Simple Summary

Prostate cancer is one of the leading causes of cancer-related deaths in men, with limited treatment options once it progresses to the advanced stage. A major reason for poor outcomes and resistance to therapy is the tumor microenvironment. This review highlights how different cells and signals within the tumor environment work together to suppress the immune system and drive disease progression/therapy resistance. We also discuss new therapeutic strategies designed to overcome these barriers, including drugs that target immunosuppressive cells, block key signaling pathways, or reprogram the tumor microenvironment to make immunotherapies more effective. By combining these approaches with biomarker-guided patient selection, we can gain deeper insights into the mechanisms driving therapy response and develop strategies that deliver more durable outcomes for men with advanced prostate cancer.

## 1. Introduction

Prostate cancer (PCa) is the second-most diagnosed malignancy and the fifth-leading cause of cancer-related deaths in men worldwide [1]. While most patients initially present with localized disease and receive appropriate treatment, a subset will either present de novo with metastatic disease or eventually progress to this stage [2]. Androgen deprivation therapy (ADT) and androgen signaling inhibitors remain the backbone of systemic treatment and are initially effective, but responses are rarely durable, leading to the development of metastatic castration-resistant prostate cancer (mCRPC), a lethal phenotype with a median survival of approximately two years [3,4]. Although multiple therapeutic agents with distinct mechanisms of action are now available, clinical benefit remains limited to subsets of patients, and resistance inevitably emerges, contributing to significant morbidity and mortality. A key determinant of this resistance is the complex and heterogeneous tumor microenvironment (TME), which promotes tumor immune evasion and fosters therapeutic failure through interactions between stromal, immune, endothelial, and neural crest-derived cells that secrete pro-tumorigenic and immunosuppressive factors.

Prostate cancer is frequently considered an immunologically “cold” tumor, characterized by a low mutational burden, limited infiltration of effector immune cells, and dominance of immunosuppressive pathways [2,5]. This reduced immunogenicity contributes to suboptimal recognition and elimination of cancer cells, driving disease progression and limiting the efficacy of systemic immunotherapy. Despite these barriers, the approval of sipuleucel-T and pembrolizumab (for MSI-high tumors) has signaled a new era of immunotherapy in PCa [3,6,7]. However, broader success requires a deeper understanding of the mechanisms of immune evasion within the TME, including the adaptive modulation of signaling pathways, induction of anti-apoptotic proteins, and reprogramming of cellular components [8,9].

**Figure 1:**
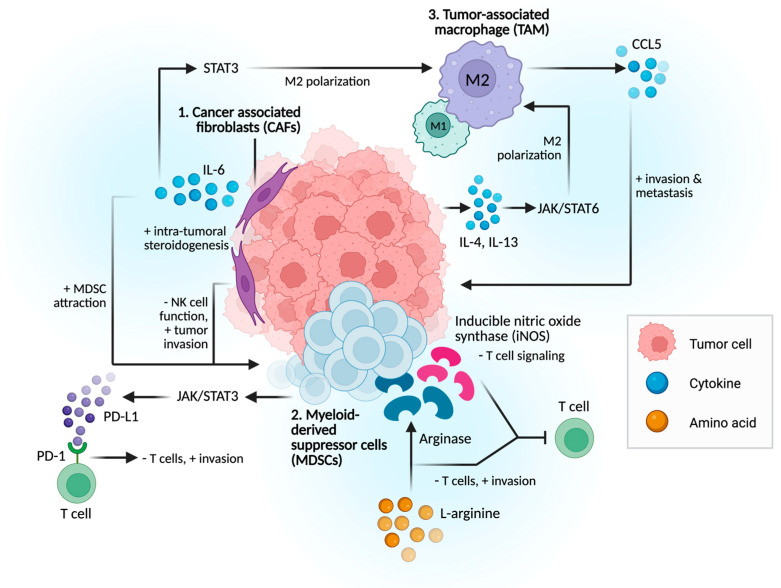
Proposed Mechanisms of Immune Evasion in Prostate Cancer. 1. CAF secretes IL-6, leading to immune suppression and treatment resistance. 2. MDSCs suppress T-cell activity through the production of arginase, iNOS, and reactive oxygen species, as well as via direct cell–cell interactions, such as PD-1/PD-L1 engagement. 3. TME cytokines and tumor-derived extracellular vesicles drive TAM polarization into immunosuppressive phenotype, leading to tumor cell migration, metastasis, and therapy resistance.

The TME is a dynamic ecosystem composed of stromal cells, immune populations, signaling molecules, vasculature, and extracellular matrix (ECM), which together influence tumor initiation, progression, and therapy response. Hot TMEs contain abundant cytotoxic T lymphocytes (CTLs) and pro-inflammatory signatures that favor immunotherapy response, whereas cold TMEs, such as that of PCa, lack sufficient immune infiltration and are dominated by suppressive mechanisms [2,5]. Immunosuppressive components include regulatory T-cells (Tregs), regulatory B cells (Bregs), inhibitory tumor-associated macrophages (TAMs), myeloid-derived suppressor cells (MDSCs), and cancer-associated fibroblasts (CAFs) (Figure 1). These cells suppress effector immunity through mechanisms such as IL-10 and TGF-β secretion, nutrient depletion, immune checkpoint ligand expression, and ECM remodeling [10,11]. In contrast, effector components including CD8+ CTLs, CD4+ Th1 helper T-cells, natural killer (NK) cells, dendritic cells (DCs), and pro-inflammatory TAMs eliminate tumor cells through antigen presentation, cytotoxic molecules such as perforin and granzymes, and pro-inflammatory cytokines, including IFN-γ and TNF-α [11]. Ultimately, tumor progression or immune control is dictated by the balance between these suppressive and effector forces within the TME.

This review focuses on the myeloid cell compartment of the TME, including TAMs, MDSCs, dendritic cells, and neutrophils. Although regulatory lymphoid cells such as Tregs and Bregs are also central to tumor immune evasion, their detailed discussion falls outside the scope of this work. By narrowing to the myeloid lineage, we provide a deeper examination of their distinct mechanisms and therapeutic potential, while acknowledging that lymphoid regulation warrants its own dedicated reviews.

## 2. Mechanisms of Immune Evasion in the Prostate Cancer Tumor Microenvironment

### 2.1. Immunosuppressive Cells and Signaling Pathways

#### 2.1.1. Cancer-Associated Fibroblasts (CAFs)

CAFs are a dominant and critical stromal cell type within the TME, where they play a central role in driving cancer progression. They promote tumor growth by remodeling the ECM to facilitate invasion, secreting pro-tumorigenic factors such as IL-6 and neuregulin 1 (NRG1) that enhance proliferation and therapy resistance, as well as fostering an immunosuppressive “cold” microenvironment. This niche is reinforced through the recruitment of MDSCs and TAMs, which suppress anti-tumor T-cell activity [12].

In PCa, fibroblasts and smooth muscle cells are key stromal components in both localized disease and mCRPC. Among these, CAFs are the most abundant and functionally diverse. They express markers such as fibroblast activation protein (FAP) and secrete mediators, including CXCL12 and IL-6, which promote tumor growth, invasion, castration resistance, and bone metastasis while shaping immune infiltration by attracting immunosuppressive cells like MDSCs or preventing the recruitment of cytotoxic T-cells [13,14,15].

Importantly, CAFs are not a uniform population but are highly heterogeneous and plastic, comprising subtypes with divergent and sometimes opposing functions. Myofibroblastic CAFs remodel the ECM to drive invasion and metastasis, while antigen-presenting CAFs may induce immune tolerance through tumor antigen presentation. Immune-regulatory CAFs enhance tumor invasiveness by interacting with MDSCs and macrophages to suppress NK cell function [14,16]. Spatial transcriptomics further highlights regional specialization, showing that tumor-central CAFs support hypoxia and metabolic reprogramming, whereas peripheral CAFs display stress and inflammatory features resembling pre-neoplastic lesions [4].

This functional diversity reflects the ability of CAFs to co-evolve with tumors in response to microenvironmental cues, ultimately driving therapy resistance. Yet, CAF plasticity also presents therapeutic opportunities: while pro-tumorigenic subtypes such as ECM CAFs, regulated by YAP1, accelerate cancer progression, anti-tumor subtypes such as Lym CAFs, driven by NF-κB signaling, can recruit and activate CD8^+^; T cells to restore immune surveillance [17,18]. Because of their ability to both support and suppress tumors, CAFs represent a compelling therapeutic target to shift the TME from tumor-promoting to tumor-restraining. This functional plasticity is especially important in the context of therapy resistance.

One key mechanism by which CAFs drive therapy resistance is through cytokine-mediated signaling. IL-6, for instance, promotes androgen insensitivity and intra-tumoral steroidogenesis [19]. An in vitro study by Neuwirt et al., 2020 [20] demonstrated that CAF-conditioned media containing high IL-6 levels was associated with enhanced cholesterol uptake and steroid production in prostate cancer cells under androgen-deprived conditions. Similarly, Patel et al., 2018 [21] linked IL-6 signaling to metabolic reprogramming that enables tumor growth despite ADT. Beyond cytokines, fibroblasts contribute directly to hormone therapy resistance. Zhang et al., 2020 [22], using prostate cancer organoids, found that when cancer cells were separated from fibroblasts, they developed resistance to bicalutamide much more slowly and never developed resistance to enzalutamide. In contrast, co-culture with fibroblasts accelerated resistance acquisition. The key factor that was implicated was NRG1, which activated the HER3/AKT signaling pathway [22]. High HER3 expression in advanced prostate cancer is correlated with a faster progression to treatment resistance and reduced overall survival [23]. NRG1 secreted by CAFs acts in a paracrine manner to sustain tumor growth and confer resistance to both enzalutamide and ADT through AR-independent mechanisms, contributing to castration resistance [22].

CXCL12 is a chemokine secreted by stromal and pro-inflammatory macrophages in prostate cancer that binds CXCR4 on lymphocytes, tumor cells, and myeloid populations to guide their migration. Within the TME, it serves as a central regulator of immune cell positioning, tumor–immune interactions, and disease progression. In PCa, elevated CXCL12 activity establishes chemotactic gradients that draw not only CD8^+^; effector T-cells but also Tregs into shared niches [10]. This spatial arrangement allows Tregs to suppress CD8^+^; cells through IL-2 sequestration and STAT5/TOX-mediated exhaustion, sustaining an immunosuppressive “cold” TME despite immune infiltration. Clinically, heightened CXCL12/CXCR4 signaling correlates with faster progression, worse prognosis, and resistance to immunotherapy [10]. Therapeutic blockade of the CXCL12/CXCR4 axis with CXCR4 inhibitors in prostate cancer models reduces Treg infiltration, restores CD8^+^; cytotoxicity, and suppresses tumor growth, with the strongest effects seen when combined with IL-2 supplementation. In vitro co-culture assays further confirm that CXCL12 driven Treg recruitment impairs CD8^+^; function through IL-2 depletion, and that replenishing IL-2 reverses this suppression. Because of this dual role, the CXCL12/CXCR4 axis is a promising therapeutic target in reprograming the TME toward an immune-active state [10].

The evolving heterogeneity of CAFs reflects their dynamic role in prostate cancer progression and therapeutic resistance. Their ability to modulate immune responses, rewire metabolic pathways, and support AR-independent survival positions them as both biomarkers and therapeutic targets. Strategies that disrupt CAF-driven resistance, such as inhibition of IL-6, hold promise for improving treatment outcomes. A deeper understanding of the molecular crosstalk between CAFs and tumor cells is essential for the development of more effective, targeted therapies in advanced prostate cancer.

#### 2.1.2. Myeloid-Derived Suppressor Cells (MDSCs)

MDSCs are a heterogeneous population of immature myeloid cells that play a pivotal role in establishing an immunosuppressive TME in PCa. Their expansion is driven by chronic inflammation and tumor-derived factors, increasing as the disease progresses to mCRPC. MDSCs suppress T-cell activity through multiple mechanisms, including the production of arginase, iNOS, and reactive oxygen species (ROS) [16,24]. Beyond their immunosuppressive function, they demonstrate remarkable plasticity, differentiating into endothelial cells, fibroblasts, TAMs, or osteoclasts, thereby contributing to tumor progression and bone metastasis [2,4,25,26,27]. The two main subsets of MDSCs are granulocytic or polymorphonuclear (PMN-MDSCs) and monocytic (M-MDSCs).

PMN-MDSCs are the more abundant subset and accumulate in the TME through a coordinated network of growth factors, cytokines, and chemokines. Expansion is driven by GM-CSF and G-CSF, while recruitment is mediated by the CXCL/CXCR1/2 and CCL2/CCR2 axes [28]. Resembling neutrophils, they exert potent immunosuppressive effects primarily through STAT3-dependent signaling [27,28]. PMN-MDSCs suppress T-cell proliferation via arginase activity, generate ROS and neutrophil elastase that impair T-cell function, and facilitate tumor invasion. In PCa, they secrete IL-23, which activates the STAT3–RORγ pathway to maintain androgen receptor signaling under androgen-deprived conditions, thereby driving castration resistance [29]. Clinically, elevated circulating PMN-MDSCs are strongly associated with poor prognosis and reduced survival in mCRPC, establishing them as a negative prognostic indicator [25,26,27].

M-MDSCs, though less abundant, also play a critical role in PCa. Their development begins in the bone marrow, where factors such as GM-CSF, G-CSF, and TNF-α stimulate sustained myelopoiesis, producing immature myeloid cells that fail to differentiate and instead acquire immunosuppressive properties [30]. Once in circulation, M-MDSCs are recruited primarily through the CCL2/CCR2 axis, supported by CCL3 and CCL4, with cancer-associated fibroblasts amplifying this process by secreting CCL2 [28,30]. Within the TME, inflammatory mediators such as IFN-γ and IL-1β activate STATs, NF-κB, and HIF-1α pathways [25,26,27], while GM-CSF promotes their differentiation into TAMs. Functionally, M-MDSCs inhibit T-cell responses through iNOS derived nitric oxide, IDO mediated tryptophan depletion, and secretion of IL-10 and TGF-β, reinforcing a self-sustaining suppressive network [28,29,30]. They also produce NRG1, which activates HER3/AKT signaling, sustaining tumor growth and conferring resistance to ADT and next-generation anti-androgens such as enzalutamide, independent of AR signaling [25,26,27].

Importantly, M-MDSC development is not governed by a single pathway but by a redundant cytokine and chemokine network. While IL-6 and GM-CSF are consistently highlighted, with IL-6 activating JAK/STAT3 and GM-CSF supporting survival, additional mediators including IL-1β, IL-10, VEGF, granulocyte colony stimulating factor, IL-23, CCL2, CXCL5, and IL-8 contribute. Prostate cancer cell-derived conditioned medium drives monocytes toward an MDSC-like phenotype more effectively than any single cytokine [27]. This redundancy explains the limited efficacy of therapies targeting single pathways. For instance, STAT3 inhibition reduces GM-CSF but does not suppress IL-6 due to compensatory signaling. Consequently, newer strategies are shifting toward broader, combination-based approaches such as cabozantinib, which targets VEGF, MET, and AXL, or regimens combining STAT3 inhibitors, CXCR2 antagonists, and checkpoint blockade.

The significance of MDSCs in PCa has been validated in preclinical models. In 2002, Wang et al. [31] showed that PTEN knockout mice, which develop PCa with complete penetrance, exhibit increased MDSC abundance. These cells upregulate IL-10, suppressing dendritic cells and macrophages, and secrete IL-23, which induces AR gene transcription through the STAT3–RORγ pathway, potentially contributing to ADT resistance. Similarly, Hellsten et al., 2019 [27] demonstrated that activation of the pSTAT3 pathway drives MDSC expansion via GM-CSF secretion, with MDSCs releasing IL-6, IL-1β, and IL-10 to reinforce immune suppression.

Beyond their mechanistic roles, MDSCs are emerging as promising biomarkers in PCa. In mCRPC, PMN-MDSC levels are significantly higher compared with non-metastatic or hormone-sensitive disease. Patients with elevated PMN-MDSC counts have markedly shorter survival (median 159 days) than those with low levels (768 days). Overall, MDSCs are central to fostering an immunosuppressive TME, promoting therapy resistance, and serving as negative prognostic biomarkers, highlighting their potential as therapeutic targets to enhance cancer immunotherapy [25,26,27].

#### 2.1.3. Tumor-Associated Macrophages (TAMs)

Macrophage polarization is a key regulator of the TME and plays a critical role in PCa progression. In PCa, TAMs predominantly adopt an immunosuppressive phenotype, helping to facilitate tumor cell migration, metastasis, and therapy resistance. Among these, the CD163_+_; (formerly referred to as M2) subset is particularly associated with aggressive disease and poor prognosis [12,32].

**Figure 2:**
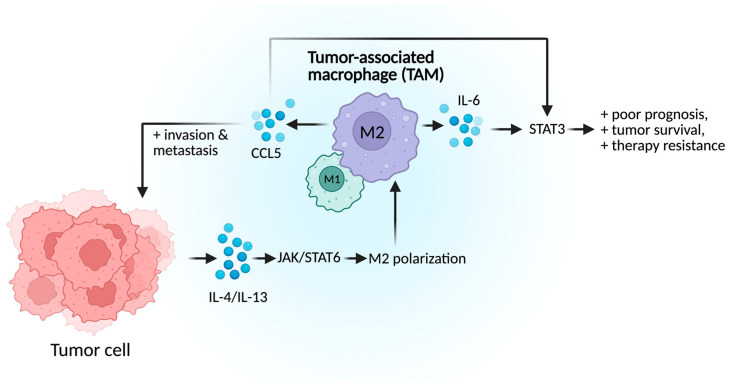
TAM-related mechanisms in PCa tumor progression. Tumor cells drive immunosuppressive (M2) macrophage polarization via IL-4/IL-13, leading to IL-6–STAT3 signaling that promotes tumor survival and therapy resistance, while CCL5 release enhances invasion and metastasis.

Macrophage polarization is driven by various TME-derived factors, including cytokines and tumor-derived extracellular vesicles. The immunosuppressive phenotype polarization is regulated by STAT3 and STAT6 activation. Pro-inflammatory cytokines, IL-4 and IL-13, trigger the JAK/STAT6 signaling cascade which facilitates IRF4 nuclear translocation and transcription of M2-associated genes such as Arg1 (Figure 2) [33]. In parallel, IL-6 secreted by TAMs activates STAT3, which leads to further immune suppression and correlates with poor prognosis, high Gleason scores, elevated PSA levels, and therapeutic resistance. TAMs also promote PCa progression through CCL5 secretion, a chemokine implicated in metastasis and cancer stemness. Immunofluorescence has demonstrated co-localization of CCL5 with CD163^+^ macrophages in both primary tumors and metastatic lymph nodes [34]. Functionally, CCL5 enhances PCa cell invasion and supports prostate cancer stem cell self-renewal in vivo [33]. Treatment of PCa cells with CCL5 promotes phenotypic changes, such as epithelial–mesenchymal transition (EMT), along with STAT3 upregulation, phosphorylation, and nuclear translocation, highlighting the CCL5-STAT3 axis as a driver of tumor aggressiveness. Knockdown of CCL5 in TAMs downregulated key metastasis and stemness markers such as STAT3. Moreover, inhibiting CCL5 significantly reduced TAM-induced PCSC self-renewal in vivo, highlighting CCL5 and STAT3 as promising therapeutic targets for suppressing PCa metastasis and treatment resistance [33,34].

### 2.2. Modulation of Immune Checkpoints

#### 2.2.1. PD-1/PD-L1

**Figure 3:**
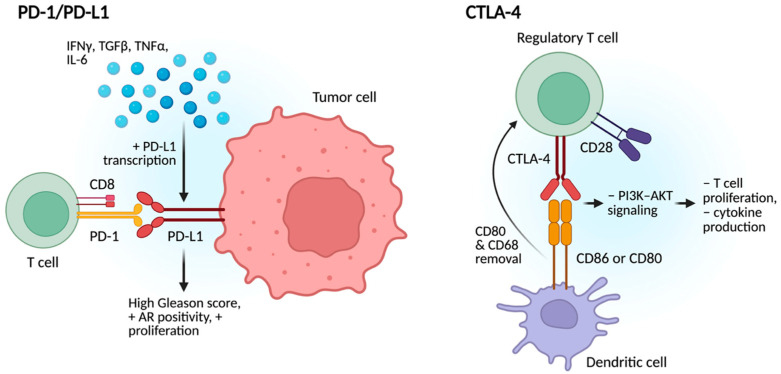
PD-L1 and CTLA-4 immune pathways in the TME. PD-1/PD-L1 signaling promotes T-cell exhaustion, NK cell suppression, and recruitment of immunosuppressive cells, while CTLA-4 enhances Treg-mediated immune evasion by blocking T-cell co-stimulation, leading to an immunosuppressive environment.

The TME plays a central role in regulating PD-L1 expressions in PCa, enabling immune evasion through the actions of immune and stromal cells, cytokines, and soluble factors [35] (Figure 3). Cytokines such as IFNγ, TGFβ, TNFα, and IL-6, as well as hypoxia, upregulate and promote PD-L1 expression via JAK/STAT3 signaling [36]. Higher PD-L1 expression contributes to T cell exhaustion by binding PD-1 and blocking T-cell receptor signaling, leading to inhibition of downstream pathways that render CD8+ T-cells ineffective. PD-L1 also impairs antigen presentation and suppresses NK cells. Additionally, PD-L1 promotes recruitment of immunosuppressive cells including Tregs, immunosuppressive macrophages, and MDSCs to further reinforce immune suppression. PD-L1 also signal within tumor cells to enhance proliferation, survival, and autophagy inhibition, potentially contributing to resistance to checkpoint inhibitors [35,36].

Clinically, PD-L1 expression is associated with aggressive features such as high Gleason score, AR positivity, and increased proliferation [37]. Over half of aggressive primary tumors show moderate-to-high PD-L1 levels. Higher PD-L1 correlates with shorter biochemical recurrence (BCR)-free survival and worse outcomes, especially in high-risk patients and those receiving hormonal therapy. PD-L1 positivity is linked to an increased risk of distant metastasis [37].

#### 2.2.2. CTLA-4

In prostate cancer, CTLA-4 is a critical immune checkpoint receptor that contributes to immune evasion by suppressing T-cell activation. It is constitutively expressed on regulatory T-cells (Tregs) and upregulated on activated effector T-cells, particularly in response to chronic antigen exposure within TME; prostate tumors frequently exhibit an accumulation of Tregs expressing high levels of CTLA-4, which enhances local immunosuppression [38]. CTLA-4 has a higher affinity than CD28 for binding the co-stimulatory molecules CD80 and CD86 on antigen-presenting cells (APCs), outcompeting CD28 and thereby disrupting the second signal required for full T-cell activation [39]. This blockade impairs PI3K–AKT signaling, leading to reduced T-cell proliferation, cytokine production (including IL-2 and TNF-α), and survival, often resulting in T-cell anergy or exhaustion [29]. CTLA-4 also facilitates the removal of CD80 and CD86 from the APC surface, further limiting T-cell co-stimulation. Although CTLA-4 is not typically mutated in prostate cancer, its immunoregulatory functions indirectly support tumor progression by weakening anti-tumor immunity, promoting the release of pro-tumorigenic factors, and fostering a microenvironment conducive to angiogenesis, invasion, and metastasis [16,39,40].

### 2.3. Metabolic Alterations

#### 2.3.1. Lactate Production

**Figure 4:**
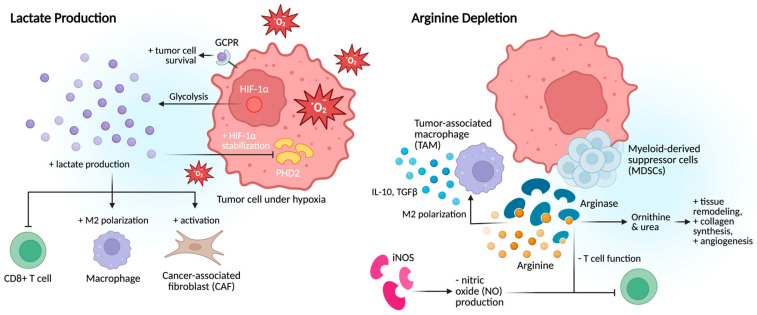
Metabolic drivers of immune evasion in prostate cancer TME. Hypoxia-driven lactate production suppresses T-cell function, stabilizes HIF-1α, and promotes angiogenesis and therapy resistance, while arginine depletion via arginase and iNOS activity impairs CD8^+^; T-cells and enhances immunosuppressive macrophage polarization.

Hypoxia within the prostate TME drives increased lactate production, which directly suppresses T-cell function and alters amino acid metabolism, depriving immune cells of essential nutrients required for effective antitumor responses [41]. Lactate is therefore not just a metabolic byproduct but a central mediator of tumor progression. It promotes migration, angiogenesis, immune evasion, extracellular matrix remodeling, and therapy resistance (Figure 4). Beyond its metabolic role, lactate acts as a signaling molecule through G-protein-coupled receptors to enhance tumor cell survival, immune suppression, and chemoresistance. It also inhibits prolyl hydroxylase domain-containing protein 2 (PHD2), leading to stabilization of HIF-1α, while modifying histones to regulate gene expression, thereby reinforcing tumor adaptability under metabolic stress [41]. Under hypoxia, lactate functions as both a fuel source and an epigenetic regulator, sustaining glycolysis and promoting angiogenesis through VEGF induction and other HIF-dependent and HIF-independent pathways.

Lactate also profoundly shapes the immune microenvironment. It drives macrophage polarization toward a pro-tumor phenotype, fuels regulatory T-cells (Tregs), and activates CAFs, collectively facilitating invasion and metastasis. By creating an immunosuppressive and metabolically reprogrammed niche, lactate promotes tumor recurrence and resistance to therapy. Targeting lactate metabolism or its downstream signaling pathways therefore represents a promising therapeutic strategy to restore antitumor immunity and improve outcomes in advanced prostate cancer.

As a biomarker, lactate reflects both glycolytic activity and tumor aggressiveness, making it valuable for monitoring disease progression and therapy response. Multiparametric MRI (mpMRI) provides precise anatomical localization of suspicious lesions, which can guide multinuclear MR spectroscopy (MRS) for direct metabolic assessment of lactate. Advanced methods such as dynamic nuclear polarization (DNP) with ^13^C-labeled pyruvate enable visualization of the Warburg effect by measuring elevated pyruvate-to-lactate conversion in cancerous tissue [42]. MRS also reveals the reverse Warburg effect, in which stromal fibroblasts generate lactate consumed by tumor cells as an energy source. These observations are further validated by ex vivo high-resolution magic angle spinning (HRMAS) spectroscopy of biopsy tissues, confirming lactate as a robust marker of malignant metabolism, reprogramming, and treatment response. Thus, integrating mpMRI with multinuclear MRS offers a powerful approach to characterizing the prostate TME through lactate metabolism [42].

#### 2.3.2. Arginine Depletion

Arginine metabolism contributes significantly to immunosuppression in PCa, primarily through the activity of arginase (ARGI and ARGII) and inducible nitric oxide synthase (iNOS) within the TME [24]. PCa cells, MDSCs, and immunosuppressive TAMs often overexpress arginase, leading to the conversion of arginine into ornithine and urea. This depletes extracellular arginine, impairing T-cell function, particularly cytotoxic CD8+ T-cells, which require arginine for proliferation, survival, and activation. Arginine metabolism also shapes the TME by promoting immunosuppressive macrophage polarization, which further expresses high levels of arginase [24]. This supports tumor growth through tissue remodeling, collagen synthesis, and angiogenesis, driven by ornithine-derived polyamines and proline. Arginine and its metabolites can also modulate gene expression and cellular metabolism in T-cells and other immune cells, contributing to their exhaustion or tolerance. Immunosuppressive macrophages reinforce this environment by secreting cytokines such as IL-10 and TGFβ, further suppressing effector immune responses [32]. In addition, arginase and iNOS compete for the same substrate, arginine. When arginase is up-regulated, arginine availability for iNOS declines, resulting in reduced nitric oxide (NO) production. Since NO is critical for T-cell- and macrophage-mediated cytotoxicity, its depletion facilitates immune evasion and tumor progression. Androgen signaling, a central driver of PCa, has also been linked to increased expression of ARGII and ARGI, promoting immunosuppression early in disease development [24].

## 3. Potential Opportunities: Modulating the Tumor Microenvironment

### 3.1. Targeting Immunosuppressive Cells

#### 3.1.1. Targeting CAFs

Human epidermal growth factor receptor 3 (HER3, also known as ERBB3) is highly expressed in prostate cancer and plays a significant role in modulating the tumor microenvironment. Although in vitro inhibition of HER3 has shown antitumor effects, prior clinical trials using ERBB-targeting agents (including HER2 and HER3 inhibitors) failed to demonstrate substantial clinical benefit in prostate cancer [23]. These shortcomings may stem from an underappreciation of ligand-induced receptor activation and the complex paracrine interactions within the TME. For instance, Gil et al. showed that HER3 is highly expressed in advanced prostate cancer and correlates with poor clinical outcomes, despite the absence of common HER3 genomic alterations. This signaling was not driven by the tumor cells themselves but rather by infiltrating myelomonocytic immune cells, which secrete NRG1 in a paracrine fashion [23]. Traditionally, paracrine growth factors were thought to be produced by cancer or neighboring stromal cells, but this study highlights tumor-infiltrating inflammatory cells as a dominant source of NRG1, emphasizing the therapeutic potential of targeting immune cell-derived signaling pathways.

Recent preclinical evidence demonstrated that the therapeutic effect of blocking NRG1 is highly dependent on PTEN status. In PTEN wild-type prostate cancer cells, NRG1 stimulation conferred resistance to AR-targeted therapy with enzalutamide, whereas in PTEN knock-out cells, NRG1 paradoxically inhibited growth and its blockade had no effect [22,43]. Notably, inhibiting NRG1-mediated HER2/HER3 signaling with the bispecific antibody zenocutuzumab restored enzalutamide sensitivity in PTEN wild-type cells, effectively reversing the resistant phenotype. In vivo, enzalutamide suppressed tumor growth in both PTEN genotypes, but only [22,43] PTEN wild-type tumors responded to the addition of zenocutuzumab [17]. These findings suggest that NRG1 promotes AR resistance through paracrine activation of PI3K-AKT signaling in PTEN wild-type prostate cancer and highlights a compelling rationale for combining AR-targeted therapy with NRG1-HER2/HER3 inhibition in selected patient populations [17,22].

Further supporting a precision medicine approach, recent studies using both patient-derived organoids and mouse models demonstrated that combining an anti-HER3 antibody with a topoisomerase I inhibitor in the form of an antibody–drug conjugate (ADC) effectively halted tumor proliferation in HER3-high prostate cancers [23]. These ADCs simultaneously block HER3 signaling and deliver cytotoxic agents directly to HER3-expressing cells, resulting in potent and sustained antitumor responses. However, the efficacy of this approach is significantly enhanced in tumors with high HER3 expression, emphasizing the importance of biomarker-driven patient selection in future clinical trials [23]. Another promising strategy involves reprogramming CAFs into a quiescent state to suppress their tumor-promoting functions. In a 2024 study, Song et al. [18] identified YAP1 as a critical regulator of CAF activation and perineural invasion via RNA sequencing of prostate cancer tissue samples. YAP1 governs the plasticity between two distinct CAF subtypes: ECM-CAF, associated with extracellular matrix remodeling and immunosuppression, and Lym-CAF, linked to immune support. Overexpression of YAP1 in Lym-CAFs promoted their transition toward the ECM-CAF phenotype, while silencing YAP1 in ECM-CAF-activated CD8^+^; T-cells and favored a shift toward the more immune-permissive Lym-CAF state. In vivo, selective depletion of YAP1 in ECM-CAFs led to slower tumor progression, increased infiltration of CD8^+^; T-cells, and reduced collagen deposition in prostate cancer mouse models [44]. Combining YAP1 depletion with anti-PD-1 immune checkpoint blockade produced synergistic effects, further suppressing tumor growth, increasing CD8^+^; T-cell infiltration, and decreasing markers of T-cell exhaustion. Currently, there is limited clinical experience regarding safety, tolerability, and effectiveness in humans, especially in prostate cancer patients. Further research is also required to evaluate how consistent and stable this phenotype switching will be in a CAF population that is inherently highly heterogeneous and plastic [18].

#### 3.1.2. Targeting MDSC and TAMs

Therapeutic strategies aimed at reversing myeloid-driven immunosuppression in prostate cancer focus on targeting MDSCs and TAMs to restore anti-tumor immunity. In mCRPC, PMN-MDSCs contribute to resistance by secreting interleukin-23 (IL-23), which activates STAT3–RORγ signaling in tumor epithelial cells. This cascade upregulates androgen receptor (AR) activity and undermines the efficacy of ADT [27,45]. Preclinical models show that genetic deletion or pharmacologic inhibition of IL-23 restores sensitivity to ADT and AR-targeted therapies, particularly in PTEN-deficient tumors, resulting in reduced proliferation and delayed progression [45].

Tildrakizumab, an FDA/EMA-approved monoclonal antibody targeting IL-23, has demonstrated a favorable safety profile in autoimmune conditions such as moderate-to-severe psoriasis [46]. Its combination with abiraterone acetate is currently being tested in a phase I/II trial for mCRPC. While abiraterone inhibits AR signaling, tildrakizumab targets IL-23–mediated inflammation and immune resistance. This dual approach is designed to produce more durable tumor control than hormonal therapy alone, particularly in patients with resistance to conventional ADT [46].

Beyond IL-23, STAT3 itself serves as a central regulator of MDSC function and tumor progression. Inhibition of STAT3 has been shown to suppress MDSC accumulation and restore immune activity in preclinical models. Hellsten et al., 2019 [27] demonstrated that galiellalactone, a selective STAT3 inhibitor, disrupts monocyte differentiation into MDSC-like cells in response to prostate cancer cell-conditioned media. Galiellalactone also suppresses the production of immunosuppressive cytokines IL-10, IL-1β, IL-6 in monocytes, while reducing factors that drive MDSC recruitment and expansion in tumor cells such as IL-8 and GM-CSF. These findings support STAT3 inhibition as a promising strategy to reprogram the TME, although galiellalactone remains in early-stage development [27].

Alternatively, VEGF signaling has shown to promote MDSC expansion in the bone marrow and recruitment into tumors through cytokines such as GM-CSF, G-CSF, M-CSF, IL-6, and IL-1β. Khaki et al., 2022 [29] demonstrated that addition of cabozantinib decreased MDSC frequencies in the spleen and lymph nodes in a mouse model for breast cancer. Reductions in tumors were observed when combined with anti-HER2 monoclonal antibody therapy. MDSCs suppress immune responses in part through enzymes such as arginase-1, and cabozantinib significantly reduced Arg-1 mRNA expression in splenic leukocytes to ~30% of control levels with monotherapy and ~10% with combination therapy. Beyond limiting suppressive function, VEGF TKIs can drive MDSC differentiation into neutrophil-like cells with tumoricidal activity. Together, these effects diminish both the number and activity of MDSCs, thereby facilitating CD8^+^; T-cell expansion and activation [29].

Immunosuppressive TAMs help to maintain a pro-tumor TME via CSF-1/CSF-1R signaling. Preclinical models show that CSF-1R inhibition reduces TAM infiltration, disrupts M2 programming, and downregulates the CXCL12/CXCR4 axis, an important pathway for tumor cell survival and chemoresistance. Combined with docetaxel, CSF-1R blockade enhances apoptosis and suppresses proliferation, providing strong rationale for combination approaches targeting both TAMs and tumor cells [33,43,47]. Further, immunosuppressive TAMs secrete CCL5, which activates STAT3 signaling and promotes stem-like features and drug resistance in prostate cancer. In docetaxel-resistant models, inhibition of the CCL5/STAT3 axis decreases metastasis and restores chemosensitivity. Blocking either pathway reduces tumor burden in vitro and in vivo, highlighting the reciprocal interactions between TAMs and tumor cells and reinforcing the need to interrupt macrophage polarization [11,34,47].

The TAM receptor tyrosine kinases, Tyro3, Axl, and MerTK, has been found to facilitate immunosuppressive programming. These receptors promote efferocytosis and drive the release of anti-inflammatory cytokines, suppressing pro-inflammatory responses. In preclinical studies, inhibition of these receptors shifts macrophage polarization toward a pro-inflammatory phenotype and reduces immune evasion, representing another avenue to therapeutically reprogram prostate cancer TME [48].

#### 3.1.3. Targeting Metabolic Pathways

Lactate, a byproduct of aerobic glycolysis in tumor cells, plays a significant role in shaping the immunosuppressive TME landscape. High levels of lactate inhibit pro-inflammatory immune activity by acting as a metabolic brake, particularly on TAMs [49]. It promotes the polarization of macrophages to the anti-inflammatory phenotype, which secrete immunosuppressive cytokines like IL-10 and TGF-β [11]. Lactate also contributes to epigenetic regulation through histone lactylation driving gene expression patterns that favor immune suppression and tissue remodeling rather than inflammation, impairing T lymphocyte and NK cells function while concurrently promoting Tregs’ expansion [49]. Additionally, lactate alters cytokine and chemokine signaling, decreasing pro-inflammatory mediators such as IFN-γ and TNF-α, while enhancing anti-inflammatory signals [49]. The interplay between tumor-derived lactate and TAM activation is a critical factor influencing treatment outcomes in prostate cancer, particularly in tumors with PTEN and p53 deficiencies [31]. In these tumors, activation of the PI3K survival signaling drives increased aerobic glycolysis, resulting in elevated lactate production. This lactate-rich microenvironment exerts direct immunosuppressive effects by inducing histone lactylation in TAMs, shifting TAMs toward an immunosuppressive, pro-tumoral phenotype, markedly reducing their capacity to phagocytose cancer cells and blunting antitumor immunity. Consequently, tumors with high lactate levels are more resistant to ADT and ICI therapies. Targeting lactate metabolism, either through synthesis or efflux, with LDHA or MCT-1/MCT-4 inhibitors has demonstrated efficacy in vivo mCRPC cell lines and murine models. PI3K inhibitors, such as copanlisib, reduces glycolysis and lactate production in tumor cells, which in turn decreases histone lactylation in TAMs and reactivates their tumoricidal function. The enhanced phagocytosis of TAMs has been shown to improve the efficacy of ADT combined with anti-PD1 immunotherapy as well as increase the effectiveness of radiotherapy and chemotherapy, particularly docetaxel [41].

Targeting arginine in prostate cancer shows promise in preclinical studies, but several factors challenge its therapeutic value, primarily arginine’s dual role: it supports both tumor cell growth and anti-tumor immune cell function, particularly T-cell activation and proliferation. Depleting arginine to starve prostate cancer cells could simultaneously suppress T-cell responses, impairing natural immune surveillance and immunotherapy. Additionally, prostate cancer exhibits heterogeneous arginine metabolism. Some tumor cells can upregulate argininosuccinate synthase (ASS) to resynthesize arginine, making them less sensitive to extracellular arginine deprivation [24]. Cancer cells are also metabolically adaptive and may re-express ASS or exploit alternative pathways to survive arginine depletion, leading to potential resistance. Systemic arginine deprivation poses another concern, as normal cells also rely on arginine, raising the concern for unintended side effects. Furthermore, while arginine deprivation agents have shown efficacy in other cancers (GI cancers), clinical evidence remains limited for prostate cancer [24]. Finally, the hormonal context matters: androgen levels can modulate the expression of key enzymes in arginine metabolism, such as ARGII and ASS. Arginine-targeted therapies may therefore be less effective in androgen-dependent tumors, which often express higher levels of ASS and are more capable of maintaining intracellular arginine pools.

#### 3.1.4. Modulating Immune Checkpoint Inhibitors

While ICIs typically target the PD-1/PD-L1 or CTLA-4 pathways, PCa frequently leverages additional inhibitory mechanisms, making it difficult for single-agent ICI therapy to succeed. Other immune checkpoint molecules may be upregulated in PCa, allowing tumors to bypass immune inhibition even when one pathway is blocked. Furthermore, the TME can induce the expression of multiple checkpoints on T-cells and APCs in response to therapy, reinforcing immunosuppression and blunting immune reactivation. As a result, monotherapies targeting PD-1 or CTLA-4 often result only in modest anti-tumor activity [50]. This has prompted the development of combination strategies, such as dual-checkpoint blockade or pairing ICIs with other immunotherapies, but these approaches tend to increase toxicity without reliably improving outcomes in prostate cancer [36,39,49]. Prostate cancer exhibits significant genetic, molecular, and immunological diversity both between patients and within individual patients across primary and metastatic lesions. This includes variation in genetic mutations, expression of immune checkpoint ligands like PD-L1, and antigen repertoires. The heterogeneous TME can also vary widely and may evolve over time. This diversity fosters treatment resistance, as sub-clones lacking therapeutic targets may survive and drive disease progression. Such heterogeneity poses challenges for biomarker development and patient selection, as markers like PD-L1 expression often fail to capture the full complexity of immune evasion, leading to unpredictable responses to therapy.

Pembrolizumab has been approved for MSI-high mCRPC after treatment with standard of care therapies [9,37,51]. Although checkpoint inhibitors ipilimumab and nivolumab have demonstrated clinical benefit for some, most responses were transient and associated adverse events were often severe, mitigating potential clinical development in this patient population. Better patient selection and side effect mitigation through molecular profiling for MSI, DDR gene mutations, or CDK12 alterations can help target therapy to those most likely to benefit while minimizing unnecessary toxicity in those unlikely to respond. The CheckMate 650 trial demonstrated limited efficacy and significant immune-related toxicities in an unselected mCRPC population, emphasizing that non-selective use of dual checkpoint inhibitors is suboptimal [52].

#### 3.1.5. Opportunities to Change the Tumor Microenvironment: Combination Therapy

Although combination regimens involving ICIs and chemotherapy, radiation, or PARP inhibitors are under investigation, robust and durable responses remain elusive for most patients. Compared to the success of ICIs in immunologically “hot” tumors with high mutation burdens and inflamed TMEs, the modest and inconsistent response rates in PCa highlight the urgent need to overcome its immunologically “cold” and suppressive environment [8,50]. We should not look at the TME as simply a bystander to the progression of prostate cancer and recognize its active role in aiding prostate cancer tumorigenesis. Further clinical focus should be on how to prime prostate cancers to become more immunogenic through combination therapies that target the TME.

#### 3.1.6. Chemotherapy

Docetaxel exerts immunomodulatory effects in addition to its cytotoxic properties. Mechanistically, it induces DNA damage in tumor cells, prompting the cytosolic release of both mitochondrial and genomic DNA, which activates the cGAS/STING pathway [34,47]. This leads to type I interferon signaling, including IFN-β production and the upregulation of interferon-stimulated genes involved in immune cell recruitment and activation. In prostate cancer, transcriptomic and histological analyses show that docetaxel, particularly when combined with ADT, increases tumor infiltration by lymphocytes, especially CD8+ T-cells. These immune effects are not observed with ADT alone. Docetaxel also upregulates PD-L1 on tumor cells and PD-1 on infiltrating lymphocytes, suggesting an adaptive resistance mechanism that may enhance sensitivity to checkpoint blockade. It further reshapes the TME by increasing CD8+ T-cell presence while reducing Tregs, with minimal changes observed in B cells, natural killer cells, or macrophages. This points to a predominantly T-cell-focused immunologic shift. Functional studies demonstrate increased T-cell receptor clonality and higher expression of activation markers following treatment. Preclinical models confirm that these immune changes improve tumor control, especially when docetaxel is combined with PD-1 or PD-L1 inhibitors. Clinically, a small retrospective study supports these findings, with patients receiving docetaxel plus the PD-1 inhibitor tislelizumab experiencing longer PSA progression-free survival compared to immunotherapy alone. Taken together, these data support the role of docetaxel as an immune sensitizer in prostate cancer, capable of transforming immunologically cold tumors into more inflamed and immune-responsive states and providing a rationale for chemo-immunotherapy combinations [53].

#### 3.1.7. Tyrosine Kinase Inhibitor (TKIs)

As single agents, VEGFR TKIs have shown limited efficacy in unselected prostate cancer populations, and their clinical use is often constrained by toxicity [54]. Many tyrosine kinases, especially those in the TAM family (Tyro3, Axl, Mer), VEGF receptors, and MET facilitate in creating an immunosuppressive microenvironment. Preclinical mouse models have been instrumental in elucidating the biological impact of TAM kinase inhibition in prostate cancer. Immunocompetent murine models have demonstrated that Axl inhibition increases infiltration of NK cells and CD8+ T-cells into the TME, enhancing responsiveness to ICIs. In vitro studies using prostate cancer cell lines similarly show that inhibition of Axl, Mer, and Tyro3 suppresses proliferation and invasion while promoting apoptosis [54]. Inhibition of TAM kinases reduces the recruitment and function of suppressive cells like Tregs and immunosuppressive macrophages and helps promote infiltration and activity of cytotoxic T-cells. This makes tumors more likely to respond to immunotherapy. Prior attempts to incorporate ICIs into mCRPC management, such as ICI monotherapy or dual-ICI regimens, have yielded only modest benefits or often limited by high toxicity and low response rates in unselected populations. These promising preclinical results have led to clinical trials evaluating tyrosine kinase inhibitors in combination with ICIs.

One approach under investigation involves combining cabozantinib, a multi-targeted tyrosine kinase inhibitor, with atezolizumab, a PD-L1 inhibitor. Cabozantinib exerts its immunomodulatory effects by targeting several pathways involved in immune evasion, including the TAM kinase family (Tyro3, Axl, Mer), VEGF, and MET signaling [48]. The results of the CONTACT-02 phase 3 study carry important implications for management of mCRPC, particularly those with extra-pelvic nodal or visceral metastases who have progressed following prior hormonal therapy [48,55]. The study demonstrated that the combination of cabozantinib and atezolizumab significantly improved rPFS compared to a second hormone therapy, offering a non-chemotherapy alternative for this high-risk population. The most pronounced benefits were observed in patients with liver metastases (6.0 vs. 2.1 months) and those previously treated with docetaxel for metastatic castration-sensitive prostate cancer (8.8 vs. 4.1 months) [55]. While the combination therapy was associated with a higher incidence of treatment-emergent adverse events, the toxicity profile was manageable, with rates of severe (grade 5) adverse events comparable to those seen in the control group [55]. Although OS data remain immature, the observed PFS benefit provides a compelling rationale to consider this combination in mCRPC subgroups with visceral metastasis. Additionally, Ganta et al. reported exception responses to cabozantinib plus ICI in a subset of men with mCRPC, highlighting the promise of such combination [56].

#### 3.1.8. AKT Inhibitor

AKT signaling regulates the polarization and function of tumor-associated macrophages (TAMs), which are key components of the TME. AKT activation promotes the immunosuppressive phenotype of TAMs, and this dynamic crosstalk between tumor cells and the TME, mediated in part by AKT signaling, offers an opportunity for therapeutic targeting [57]. Resistance to AR antagonists occurs in about 20–40% of patients who progress to mCRPC often due to AR alterations and compensatory activation of survival pathways like PI3K/AKT/mTOR [58]. Reciprocal feedback between these pathways allows tumor cells to escape therapy as AR inhibition can activate AKT signaling via increased phosphorylation, while AKT blockade can stimulate AR signaling, promoting cancer progression [59]. The crosstalk between these two pathways justifies dual-pathway inhibition as a strategy to overcome resistance. PTEN loss, present in 40–60% of mCRPC cases, is a key driver of this crosstalk which leads to constitutive AKT activation. Preclinical studies show that dual therapy, such as combining ipatasertib (an AKT inhibitor) with enzalutamide, synergistically suppresses proliferation, induces apoptosis, and delays resistance [59,60,61]. Clinical data reinforce this, demonstrating improved radiographic progression-free survival (rPFS) and objective response rates in patients with PTEN loss compared to AR monotherapy. However, the adverse event (AE) profile of this combination limits its broader clinical adoption. Approximately 56% of patients experience Grade ≥ 3 AEs, including diarrhea, nausea, fatigue, rash, and metabolic complications such as hyperglycemia, which may interfere with compliance and quality of life [58].

The combination of capivasertib, a pan-AKT inhibitor, with chemotherapy docetaxel has also been explored. Sequential administration of capivasertib after docetaxel is thought to target docetaxel-resistant “persister cells” that upregulate AKT signaling to survive treatment; however, the ProCAID phase II trial show that this regimen is effective across PTEN statuses, challenging a major limitation of earlier AKT-targeted therapies [59,61]. These findings suggest that AKT inhibition is effective not only in genomically selected populations but also in a broader range of patients, especially when sequenced after chemotherapy. Docetaxel does not consistently increase p-AKT, but it enhances phosphorylation of downstream effectors, which better reflect survival signaling and therapeutic vulnerability, while Capivasertib selectively disrupts survival pathways activated by chemotherapy, particularly in residual chemo-resistant cells [61]. Altogether, these findings support a shift in how resistance and sensitization are understood. Rather than viewing chemotherapy and targeted resistance mechanisms in isolation, this research highlights their interdependence: chemotherapy induces survival adaptations that can be selectively targeted post-treatment. By eliminating these resistant populations, combination strategies may improve long-term outcomes more effectively than monotherapy [58,59].

#### 3.1.9. Radioligand Therapy

Radiotherapy remodels the prostate cancer TME by both enhancing antitumor immunity and inducing compensatory immunosuppressive mechanisms, which has important implications for combination strategies with immunotherapy [62]. Lutetium-177, a prostate-specific membrane antigen (PSMA) targeted radioligand therapy, is now a standard of care for the treatment of mCRPC. The phase III VISION trial demonstrated that adding PSMA RLT to standard of care significantly improved overall survival (15.3 vs. 11.3 months) and radiographic progression-free survival (8.7 vs. 3.4 months) with a manageable side effects profile that included dry mouth and fatigue [63,64]. This represents a timely opportunity for combination approaches targeting the TME, and ongoing trials with immunotherapy, ARPIs, and DNA damage response inhibitors will determine its clinical utility [65].

#### 3.1.10. PARP Inhibitors

PARP inhibitors exert both immunostimulatory and immunosuppressive effects on the prostate cancer tumor microenvironment, enhancing antitumor immunity while also potentially promoting immune escape via PD-L1 upregulation [66]. PARPi selectively targets HRR-deficient tumor cells, leading to the accumulation of unrepaired DNA damage while sparing normal tissue. These agents offer significant benefit to men with mCRPC who harbor mutations in DNA damage response (DDR) or homologous recombination repair (HRR) genes, both as monotherapy or in combination with ARPI [67,68,69].

Despite its promise, several combination strategies involving PARP inhibitors and other therapies like ICI’s, TKIs, AKT inhibitors, or radioligands, have shown limited benefit and high toxicity. While preclinical data suggested potential synergy, especially through increased tumor immunogenicity, clinical results have been disappointing, with low response rates and frequent severe side effects. Any observed benefit was mostly confined to patients with HRR deficiencies, and even then, the added value of PARPi is outweighed by the increased adverse events [70,71]. Further development of other combinations and better patient selection are key to its success going forward.

#### 3.1.11. Microbiome

The microbiome alters the prostate cancer tumor microenvironment by promoting chronic inflammation, modulating immune cell infiltration and polarization, influencing androgen metabolism, and driving epigenetic changes [72]. Dysbiosis has been increasingly implicated in PCa progression and therapeutic response. Alterations in the gut or genitourinary microbiome can promote chronic inflammation, compromise the prostate epithelium, and trigger immune infiltration, oxidative stress, and DNA damage. Microbes like Escherichia coli and Cutibacterium acnes activate pro-inflammatory pathways and are frequently found in malignant prostate tissues [73]. High-fat diets can shift microbial composition and increase metabolites like butyrate, which may promote tumor growth. In mouse models, fecal microbiota transfer from patients with castration-resistant prostate cancer accelerated tumor progression, partly driven by bacteria like Ruminococcus, which are associated with upregulation of oncogenes [73].

The microbiome has been implicated to modulate treatment efficacy. In preclinical studies, microbiome-targeted interventions, including antibiotics, probiotics, and fecal microbiota transplant, have delayed resistance and improved outcomes [74]. Similarly, the efficacy of immune checkpoint inhibitors ICIs has been linked to the presence of beneficial gut microbes such as Bifidobacteriaceae and Akkermansia, while dysbiosis is associated with lower response rates [73]. ADT alters gut microbial composition, enriching species like Akkermansia muciniphila, which may enhance anti-tumor immunity. Chemotherapy outcomes may also be compromised by reduced microbial diversity following repeated antibiotic exposure, which can dampen immune surveillance and promote tumor-supportive metabolic pathways [74].

There is limited understanding of what and how microbes affect metabolite production, immune modulation, or other pathways. Conflicting results, especially those involving organisms like *E. coli* or *C. acnes*, likely reflect technical variability or contamination, especially in low biomass samples. Most of the current literature is based on small, cross-sectional studies. Environmental influences like diet, antibiotics, obesity, and host immunity further complicate the picture. Standardization across sampling, sequencing, and analysis methods is still lacking, which makes data difficult to compare across studies [73].

#### 3.1.12. Other Combinations

Lactate plays several key roles within the TME, including acidifying the local milieu and suppressing antitumor immunity through epigenetic reprogramming. In TAMs, lactate drives histone lactylation, which impairs their ability to phagocytose and eliminate tumor cells. Recent studies have explored a combinatorial approach that targets this axis using metabolic inhibitors such as copanlisib, a PI3K inhibitor, and ipatasertib, an AKT inhibitor [41]. By blocking signaling pathways that drive glycolysis and lactate production, these agents reduce histone lactylation in TAMs and promote a shift toward a more activated, pro-inflammatory phenotype [49]. ADT complements this strategy by increasing TAM infiltration and priming them for further activation. ICIs targeting PD-1 or PD-L1 then release inhibitory signals not only on T-cells but also on TAMs, enhancing their cytotoxic potential once the suppressive metabolic barrier is lifted. This combination of metabolic inhibition, ADT, and checkpoint blockade addresses multiple layers of immune suppression. Monotherapy or dual therapy often falls short due to compensatory mechanisms such as Wnt/β-catenin signaling, which can restore lactate production and sustain immune evasion [75]. The goal of this multi-pronged approach is to reprogram the immune microenvironment, restore effector cell function, and achieve durable tumor control.

Preclinical studies in PTEN/p53-deficient prostate cancer models support this strategy. Triple therapy significantly improves tumor control, with response rates approaching 60 percent, whereas single or double-agent regimens are substantially less effective. Nevertheless, resistance can emerge through escape pathways like β-catenin reactivation, underscoring the adaptive complexity of immune evasion. Clinically, the IPATential150 trial evaluated ipatasertib with abiraterone in metastatic castration-resistant prostate cancer with PTEN loss and showed modest improvements in progression-free survival [76]. Similarly, the CAPItello-281 trial is assessing capivasertib with abiraterone in metastatic hormone-sensitive disease. Concurrently, early-phase trials are investigating combinations of PD-1 or PD-L1 inhibitors with PI3K or AKT blockade and androgen-targeted therapies, particularly in high-risk subtypes such as PTEN-deficient tumors characterized by immune exclusion. These efforts aim to establish immunometabolic therapy as a viable strategy to convert treatment-resistant prostate tumors into immunologically active disease with the potential for durable responses [16,36,37].

#### 3.1.13. Key to Future Success

Major challenges associated with the prostate cancer tumor microenvironment evading currently available treatments include immunosuppression, cellular plasticity, stromal interactions, and metabolic adaptation. Better understanding of acquired resistance is critical, with approaches such as combining agents that suppress resistance pathways and implementing longitudinal genomic monitoring through re-biopsy or liquid biopsy. Central to these strategies is molecular profiling, which classifies tumors based on genetic, protein, and cellular characteristics. This allows clinicians to identify patients more likely to respond to targeted therapies or immunotherapies. Profiling can uncover mutations, gene losses such as PTEN, or expression of immunomodulatory proteins such as PD-L1 that influence tumor–immune interactions and therapeutic response. It also helps identify patients unlikely to benefit from certain treatments by reducing unnecessary toxicity, avoiding ineffective interventions, and optimizing resource allocation [55]. One such example is the ongoing effort to use PARPi in mCRPC with the aim to expand their use beyond patients with BRCA mutations or homologous recombination repair (HRR) defects. Investigational predictive biomarkers include RAD51 foci assays and homologous recombination deficiency signatures [77]. To improve tolerability, next-generation PARP1-selective inhibitors with reduced hematologic toxicity are in development. PARP inhibitors are also being tested in earlier disease stages, as in the AMPLITUDE and TALAPRO-3 trials in metastatic hormone-sensitive prostate cancer. Key priorities include optimizing sequencing with androgen receptor-targeted therapies or chemotherapy and identifying candidates for early treatment intensification [70].

In concordance with adaptive towards a more patient-specific model of cancer care, single-cell technologies like scRNA-seq and scDNA-seq have transformed how we approach cancer therapy by uncovering tumor heterogeneity, rare subclones, and resistant cell populations that bulk sequencing often misses [77]. These methods have helped identify key immunosuppressive components of the TME including CAFs, TAMs, and proangiogenic endothelial cells and have led to new combination strategies currently under investigation such as pairing ICIs with anti-CAF agents [23,44]. scRNA-seq has also revealed actionable stromal-derived targets like CXCL12, which are currently being explored in preclinical studies [78]. Beyond prostate cancer, single-cell profiling has uncovered hidden vulnerabilities in other cancers as well. In glioblastoma, for example, scRNA-seq identified a small population of IRS1/2-amplified cells driving resistance to dasatinib, which led to trials exploring insulin and AKT pathway inhibitors as more tailored options [79]. Single-cell analysis has also advanced our understanding of cancer stem cell populations, identifying key regulators like SOX9 and LIF/LIFR that are associated with recurrence and metastasis [80].

Single-cell analysis has expanded our view of targetable pathways by identifying new checkpoint molecules like TIGIT, PTGER4, and CCR8 on dysfunctional or suppressive immune cells. Subsets of CD73-high macrophages and specific CAF populations have also been linked to immune evasion and resistance, suggesting opportunities to reprogram the tumor microenvironment to improve the therapeutic response [80]. That said, scRNA-seq only captures mRNA expression. Spatial transcriptomics and proteomics retain tissue architecture and provide insight into how physical cell positioning and local microenvironments affect response including identifying invasive fronts or immune-excluded zones in prostate cancer [78,79]. Real-time monitoring with single-cell liquid biopsies will allow dynamic adjustments to therapy based on how a patient’s tumor is changing over time [79].

## 4. Conclusions

Prostate cancer TME significantly limits the maximal efficacy of currently available therapies through various immune evasion mechanisms. To overcome these barriers, emerging strategies aim to target immunosuppressive cells, block immune checkpoints, and modulate the TME. Key to future success of this therapeutic approach will be critically dependent on selecting the best synergistic target combinations and appropriate biomarker driven patient selection.

## Abbreviations

The following abbreviations are used in this manuscript:

PCa	Prostate Cancer
ADC	Antibody–drug conjugate
ADT	Androgen deprivation therapy
APCs	Antigen-presenting cells
AR	Androgen receptor
ASS	Argininosuccinate synthase
BCR	Biochemical recurrence
CAFs	Cancer-associated fibroblasts
mCRPC	Metastatic castration-resistant prostate cancer
dMMR	Mismatch repair deficiency
EMT	Epithelial–Mesenchymal transition
ERBB	Erythroblastic leukemia viral oncogene homolog
GM-CSF	Granulocyte-macrophage colony-stimulating factor
HIF-1α	Hypoxia-inducible factor-1 alpha
PHD2	Prolyl hydroxylase domain-containing protein 2
iNOS	Inducible nitric oxide synthase
ICI	Immune checkpoint inhibitor
IL-6	Interleukin 6
IL-10	Interleukin 10
MDSCs	Myeloid-derived suppressor cells
MSI-High	Microsatellite instability-high
OS	Overall survival
PFS	Progression-free survival
NRG1	Neuregulin-1
ROS	Reactive oxygen species
PSA	Prostate-specific antigen
PARPi	Poly (ADP-ribose) polymerase inhibitor
PSMA RLT	PSMA radioligand therapy
TAMs	Tumor-associated macrophages
TKI	Tyrosine kinase inhibitor
TME	Tumor microenvironment
PSMA	Prostate-specific membrane antigen
PMN-MDSCs	Polymorphonuclear myeloid-derived suppressor cells
HER3	Human epidermal growth factor receptor 3
Tregs	Regulatory T-cells
TMB	Tumor mutational burden
VEGF	Vascular endothelial growth factor
ARTA	Androgen receptor-targeted agents
M-MDSCs	Monocytic myeloid-derived suppressor cells
